# Association of dual SGLT-2 inhibitor and GLP-1 receptor agonist therapy with colon cancer risk in post-polypectomy patients with diabetes: a target trial emulation

**DOI:** 10.1186/s13098-026-02151-x

**Published:** 2026-03-29

**Authors:** Chih-Hsuan Wang, Wei-Cheng Chang, Hsin-Yu Chen, Po-Huang Chen, Ming Hsun Lin, Cho-Hao Lee

**Affiliations:** 1 Division of Gastroenterology, Dept. of Internal Medicine, Kaohsiung Armed Forces General Hospital, Kaohsiung, Taiwan; 2 Division of Gastroenterology, Dept. of Internal Medicine, Tri-Service General Hospital, National Defense Medical University, Taipei, Taiwan; 3 Department of Ophthalmology, Universe Eye Center, Taiwan Taipei,; 4 Department of Family Medicine, Chi Mei Medical Center, Tainan, Taiwan; 5 Division of Hematology and Oncology, Dept. of Internal Medicine, Tri-Service General Hospital, National Defense Medical University, Taipei, Taiwan; 6 Department of Oncology, Tri-Service General Hospital, National Defense Medical University, Taipei, Taiwan; 7 Division of Endocrinology and Metabolism, Dept. of Internal Medicine, Tri-Service General Hospital, National Defense Medical University, Taipei, Taiwan

**Keywords:** SGLT-2 inhibitor, GLP-1 receptor agonist, Colon cancer, Type 2 diabetes mellitus, Post-polypectomy, Target trial emulation, Chemoprevention, Propensity score matching

## Abstract

**Background:**

Patients with type 2 diabetes mellitus (T2DM) have an elevated risk of colorectal cancer. SGLT-2 inhibitors and GLP-1 receptor agonists have demonstrated potential anticancer properties through distinct mechanisms. However, the association of combination therapy with colon cancer risk remains unknown.

**Methods:**

We conducted a target trial emulation using the TriNetX Global Collaborative Network (2017–2025). Adults with T2DM and a history of polypectomy receiving dual therapy (SGLT-2 inhibitor plus GLP-1 receptor agonist) were compared with SGLT-2 inhibitor monotherapy using 1:1 propensity score matching without replacement. A new-user design and 180-day landmark analysis were employed to address immortal time bias. The primary outcome was incident colon cancer. Secondary outcomes included colectomy, other gastrointestinal cancers, cardiovascular events, renal outcomes, and all-cause mortality. Bonferroni correction was applied for multiple secondary comparisons (α = 0.05/11 = 0.0045).

**Results:**

After propensity score matching, 28,934 patients were included in each group. Dual therapy was associated with a lower risk of colon cancer compared with SGLT-2 inhibitor alone (HR 0.786, 95% CI 0.671–0.919; P = 0.003), representing a 21% relative risk reduction (NNT = 490 over 5 years). Significant reductions were also observed in colectomy (HR 0.456), other gastrointestinal cancers (HR 0.690), end-stage renal disease (HR 0.808), major adverse kidney events (HR 0.710), and all-cause mortality (HR 0.658). Negative control outcomes showed no meaningful differences, supporting study validity.

**Conclusions:**

In post-polypectomy T2DM patients, dual therapy was associated with a 21% lower risk of colon cancer versus SGLT-2 inhibitor monotherapy. Co-primary analysis demonstrated this association also persisted when comparing dual therapy against GLP-1 receptor agonist monotherapy alone (HR 0.871; P = 0.041), indicating additive chemopreventive effects of the combination warranting prospective investigation.

**Supplementary Information:**

The online version contains supplementary material available at 10.1186/s13098-026-02151-x.

## Introduction

Colorectal cancer (CRC) is the third most common cancer worldwide and the second leading cause of cancer-related mortality [1]. Patients with type 2 diabetes mellitus (T2DM) have a 1.3–1.5-fold increased risk of CRC compared with non-diabetic individuals, attributed to hyperinsulinemia, chronic inflammation, and altered gut microbiome [2, 3]. Elevated insulin and free insulin-like growth factor-1 (IGF-1) promote proliferation of colonic epithelial cells and confer survival benefits to transformed cells, ultimately facilitating colorectal carcinogenesis [4, 5]. Post-polypectomy patients represent a high-risk population in whom chemoprevention strategies could have significant clinical impact, yet no pharmacological chemoprevention is currently established.

Sodium-glucose cotransporter-2 (SGLT-2) inhibitors have emerged as antidiabetic agents with potential anticancer properties. Preclinical studies demonstrate that SGLT-2 inhibitors inhibit cancer cell proliferation by reducing glucose uptake, inducing metabolic stress, activating AMPK/mTOR pathways, promoting ferroptosis, and suppressing the Wnt/β-catenin signaling pathway implicated in colorectal carcinogenesis [6–9]. Clinical evidence supports associations between SGLT-2 inhibitor use and reduced CRC risk (HR 0.71–0.80) [10–15].

Glucagon-like peptide-1 (GLP-1) receptor agonists exert anticancer effects through reduction of obesity-related inflammation, improvement in insulin resistance, inhibition of cell proliferation, and direct anti-tumor immune effects [16, 17]. Large observational studies have reported that GLP-1 RA use is associated with a 23–30% reduction in CRC risk [18, 19]. Current guidelines from the American Diabetes Association and European Association for the Study of Diabetes recommend combination SGLT-2 inhibitor plus GLP-1 RA therapy for patients with T2DM and established cardiovascular disease or high cardiovascular risk, based on additive cardiorenal benefits [20–23].

Despite the established individual anticancer potential of both drug classes, no study has investigated whether dual SGLT-2 inhibitor plus GLP-1 RA therapy confers additional protection against colon cancer beyond either agent alone. We therefore conducted this target trial emulation study to compare colon cancer risk between patients receiving dual therapy versus SGLT-2 inhibitor monotherapy among post-polypectomy patients with T2DM.

## Methods

### Target trial emulation framework

This study employed a target trial emulation framework to estimate the effect of dual SGLT-2 inhibitor plus GLP-1 RA therapy versus SGLT-2 inhibitor monotherapy on colon cancer risk [24–27]. The target trial protocol specification is provided in eMethods Sect. 2 (Supplementary Materials).

### Data source

We utilized the TriNetX Global Collaborative Research Network, a federated real-world data platform providing de-identified electronic health record data from healthcare organizations worldwide [28, 29]. The network contained data from 196,307,143 patients across 170 healthcare organizations. Data from January 2017 through December 2025 were used.

### Study population

**Fig. 1:**
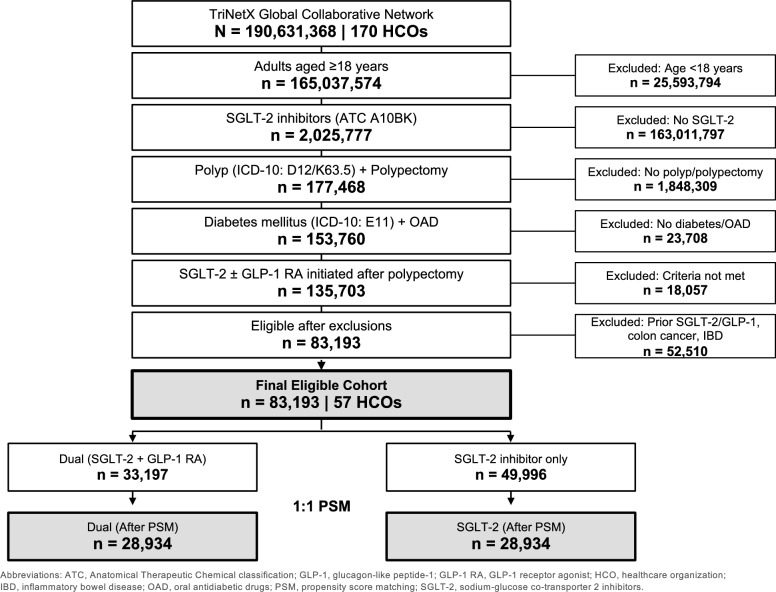
Study flow diagram showing patient selection from the TriNetX Global Collaborative Network

Eligible patients were adults aged ≥ 18 years with type 2 diabetes mellitus (ICD-10: E11.x), a history of colon polyp diagnosis (D12.x, K63.5), and a documented polypectomy procedure (CPT: 45,384–45,390), who had initiated treatment after polypectomy. Exclusion criteria included prior colorectal cancer (C18–C20), inflammatory bowel disease (K50.x, K51.x), and prior SGLT-2 inhibitor or GLP-1 RA use (new-user design). Patient selection is detailed in Fig. 1 and eTable 1 (Supplementary Materials).

### Treatment strategies and index date

Two treatment strategies were defined.

(1) dual therapy — initiation of both an SGLT-2 inhibitor and a GLP-1 RA after polypectomy, with the index date as the first date of concurrent prescription overlap; and (2) SGLT-2 inhibitor monotherapy — SGLT-2 inhibitor initiation after polypectomy with no GLP-1 RA use, with the index date as the first SGLT-2 inhibitor prescription. SGLT-2 inhibitors included dapagliflozin, empagliflozin, canagliflozin, ertugliflozin, and bexagliflozin. GLP-1 RAs included semaglutide, liraglutide, dulaglutide, exenatide, tirzepatide, and lixisenatide. Drug codes for SGLT-2 inhibitors and GLP-1 receptor agonists are provided in eTable 13.

### New-User design and landmark analysis

A new-user design restricted the population to patients without prior SGLT-2 inhibitor or GLP-1 RA exposure before the index date [30]. A 180-day landmark analysis was implemented to address immortal time bias; patients who experienced the primary outcome or died before Day 180 were excluded, and follow-up for outcome assessment began at Day 180.

### Baseline period and covariate assessment

All baseline covariates were assessed during the 1-year period preceding the index date. Covariates included: demographics (age, sex, race/ethnicity); comorbidities (essential hypertension, heart failure, ischemic heart disease, chronic kidney disease [CKD], cerebrovascular disease, overweight/obesity, dyslipidemia); concomitant antidiabetic medications (biguanides, sulfonylureas, alpha-glucosidase inhibitors, thiazolidinediones, dipeptidyl peptidase-4 inhibitors [DPP-4i], insulin); aspirin and non-steroidal anti-inflammatory drugs (NSAIDs); tobacco use disorder (F17.x, Z72.0); alcohol use disorder (F10.x, Z72.1); family history of gastrointestinal malignancy (Z80.0); and laboratory values (BMI, HbA1c, serum creatinine). A complete covariate list is provided in eMethods Sect. 7 and eTable 15 (Supplementary Materials).

### Propensity score matching

To control for measured confounding, 1:1 propensity score matching without replacement was performed using a greedy nearest-neighbor algorithm with a caliper of 0.1 standard deviation of the logit of the propensity score. Covariate balance was assessed using standardized mean differences (SMD), with SMD < 0.1 indicating adequate balance. The TriNetX platform implements propensity score matching rather than inverse probability of treatment weighting (IPTW); this approach is consistent with prior large-scale observational studies using this platform and yields estimates comparable to IPTW in large samples.

### Outcomes

The primary outcome was incident colon cancer (ICD-10: C18.0–C18.9, C19, C20) after the landmark date. Secondary outcomes included: colectomy; other gastrointestinal cancers; major adverse cardiovascular events (MACE); heart failure; end-stage renal disease (ESRD); major adverse kidney events (MAKE); acute kidney injury; all-cause mortality; acute pancreatitis; and urinary tract infection. Negative control outcomes were inguinal hernia and appendicitis. Outcome definitions are provided in eTable 14 (Supplementary Materials).

### Statistical analysis

Kaplan–Meier survival analysis estimated colon cancer-free survival in each group. Hazard ratios (HR) with 95% confidence intervals (CI) were calculated using Cox proportional hazards regression (dual therapy as reference). Risk ratios (RR) were calculated at 5-year follow-up. The number needed to treat (NNT) was calculated as the inverse of the absolute risk difference. E-values were calculated to quantify robustness to unmeasured confounding. Bonferroni correction was applied for 11 secondary outcomes (adjusted α = 0.0045).

Subgroup analyses covered age, sex, race/ethnicity, BMI, obesity status, HbA1c, concomitant medications, and GLP-1 RA type (eTables 3–7, Supplementary Materials). A co-primary analysis compared dual therapy versus GLP-1 RA monotherapy (N = 13,782 matched pairs after 1:1 propensity score matching) to assess whether the combination confers incremental benefit beyond GLP-1 RA alone. Sensitivity analyses included: (1) 1-year and 3-year follow-up; (2) age ≥ 50 years; (3) US population only; (4) 1-year landmark analysis; (5) 90-day landmark analysis (N = 27,456 per group); (6) per-protocol analysis restricted to patients with ≥ 6 prescriptions of each agent (N = 5,752 per group); (7) colonoscopy procedure rate comparison (CPT 45378–45,398) to evaluate surveillance bias; and (8) post-treatment BMI and HbA1c changes (eFigure 2, Supplementary Materials). All analyses used a two-sided alpha of 0.05.

## Result

### Study population

From the TriNetX network of 196,307,143 patients across 170 healthcare organizations, we identified adults with T2DM (E11.x) who had a colon polyp diagnosis. After applying all eligibility criteria, 83,193 patients from 57 healthcare organizations were eligible, comprising 33,197 in the dual therapy group and 49,996 in the SGLT-2 inhibitor only group. Following 1:1 propensity score matching, 28,934 patients remained in each group (Fig. 1).

### Baseline characteristics

Before propensity score matching, patients in the dual therapy group were younger (64.8 ± 9.0 vs 68.4 ± 9.6 years; SMD 0.380), more likely female (46.0% vs 39.1%; SMD 0.140), had higher BMI (34.4 ± 7.4 vs 31.5 ± 6.9 kg/m^2^; SMD 0.409) and HbA1c (8.1 ± 1.7% vs 7.5 ± 1.6%; SMD 0.359), with lower prevalence of heart failure (SMD 0.276), ischemic heart disease (SMD 0.201), and CKD (SMD 0.106), but higher obesity prevalence (SMD 0.232).

After propensity score matching, all baseline characteristics were well balanced with SMD < 0.1 for all variables, including BMI (SMD 0.095). Tobacco use (dual 11.8% vs SGLT-2i 11.8%; SMD < 0.001) and alcohol use disorder (dual 2.5% vs SGLT-2i 2.0%; SMD 0.031) were similarly balanced. Complete baseline characteristics are shown in Table 1.

**Table 1:** Baseline Characteristics Before and After Propensity Score Matching

Characteristics	Before PSM				After PSM			
	**Dual**	**SGLT-2**	***P***	**SMD**	**Dual**	**SGLT-2**	***P***	**SMD**
	(n = 33,197)	(n = 49,996)			(n = 28,934)	(n = 28,934)		
**Demographics**								
Age at Index, years	64.8 ± 9.0	68.4 ± 9.6	< 0.001	0.380	65.6 ± 8.8	65.6 ± 9.3	0.403	0.007
**Gender**								
Female, n (%)	15,276 (46.0)	19,548 (39.1)	< 0.001	0.140	12,719 (44.0)	12,511 (43.2)	0.081	0.014
Male, n (%)	17,918 (54.0)	30,445 (60.9)	< 0.001	0.140	16,212 (56.0)	16,423 (56.8)	0.077	0.015
**Race**								
White, n (%)	21,615 (65.1)	32,524 (65.1)	0.863	0.001	18,801 (65.0)	18,952 (65.5)	0.187	0.011
Black/African American, n (%)	7,138 (21.5)	10,362 (20.7)	0.007	0.019	6,184 (21.4)	6,038 (20.9)	0.137	0.012
Asian, n (%)	1,574 (4.7)	3,117 (6.2)	< 0.001	0.066	1,464 (5.1)	1,502 (5.2)	0.474	0.006
Unknown Race, n (%)	1,213 (3.7)	1,837 (3.7)	0.878	0.001	1,081 (3.7)	1,065 (3.7)	0.725	0.003
**Diagnosis**								
Essential hypertension, n (%)	26,672 (80.3)	38,797 (77.6)	< 0.001	0.067	23,066 (79.7)	22,919 (79.2)	0.130	0.013
Heart failure, n (%)	6,659 (20.1)	16,036 (32.1)	< 0.001	0.276	6,369 (22.0)	6,030 (20.8)	0.001	0.029
Ischemic heart diseases, n (%)	9,897 (29.8)	19,672 (39.3)	< 0.001	0.201	9,160 (31.7)	9,018 (31.2)	0.203	0.011
Chronic kidney disease, n (%)	8,996 (27.1)	15,973 (31.9)	< 0.001	0.106	8,098 (28.0)	7,917 (27.4)	0.093	0.014
Cerebrovascular diseases, n (%)	3,070 (9.2)	6,195 (12.4)	< 0.001	0.101	2,827 (9.8)	2,735 (9.5)	0.194	0.011
Overweight and obesity, n (%)	14,322 (43.1)	15,982 (32.0)	< 0.001	0.232	11,501 (39.7)	11,294 (39.0)	0.078	0.015
Dyslipidemia, n (%)	25,611 (77.1)	37,880 (75.8)	< 0.001	0.033	22,185 (76.7)	22,107 (76.4)	0.444	0.006
Nicotine dependence (F17), n (%)	2,313 (11.5%)	4,241 (12.8%)	0.437	0.037	1,749 (11.8%)	1,750 (11.8%)	0.248	< 0.001
Alcohol-related disorders (F10), n (%)	466 (2.3%)	958 (2.9%)	0.335	0.035	370 (2.5%)	354 (2.4%)	0.466	0.007
Family history of GI malignancy (Z80.0), n (%)	678 (3.4%)	1,684 (5.1%)	< 0.001	0.084	570 (3.9%)	596 (4.0%)	0.235	0.009
**Medication**								
Biguanides, n (%)	16,221 (48.9)	18,673 (37.3)	< 0.001	0.234	13,434 (46.4)	13,457 (46.5)	0.848	0.002
Sulfonylureas, n (%)	7,269 (21.9)	7,069 (14.1)	< 0.001	0.203	5,669 (19.6)	5,583 (19.3)	0.366	0.008
Alpha glucosidase inhibitors, n (%)	73 (0.2)	63 (0.1)	0.001	0.023	58 (0.2)	43 (0.1)	0.135	0.012
Thiazolidinediones, n (%)	1,496 (4.5)	1,154 (2.3)	< 0.001	0.121	1,038 (3.6)	993 (3.4)	0.309	0.008
Insulin, n (%)	1,714 (5.2)	1,103 (2.2)	< 0.001	0.157	1,139 (3.9)	996 (3.4)	0.002	0.026
DPP-4 inhibitors, n (%)	2,378 (11.9%)	1,851 (5.6%)	< 0.001	0.224	1,450 (9.8%)	1,430 (9.7%)	0.235	0.005
Aspirin, n (%)	3,722 (18.6%)	6,072 (18.3%)	0.335	0.008	2,757 (18.6%)	2,770 (18.7%)	0.194	0.002
NSAIDs, n (%)	1,380 (6.9%)	2,118 (6.4%)	0.121	0.021	977 (6.6%)	968 (6.5%)	0.210	0.002
**Laboratory**								
BMI, kg/m^2^	34.4 ± 7.4	31.5 ± 6.9	< 0.001	0.409	34.0 ± 7.5	33.4 ± 7.0	0.019	0.095
BMI ≥ 30 kg/m^2^, n (%)	20,738 (62.5)	24,812 (49.6)	< 0.001	0.261	17,162 (59.3)	16,996 (58.7)	0.161	0.012
BMI < 30 kg/m^2^, n (%)	9,658 (29.1)	21,228 (42.5)	< 0.001	0.282	9,300 (32.1)	9,246 (32.0)	0.630	0.004
HbA1c, %	8.1 ± 1.7	7.5 ± 1.6	< 0.001	0.359	8.0 ± 1.7	7.9 ± 1.7	< 0.001	0.048
HbA1c ≥ 7%, n (%)	22,980 (69.2)	23,593 (47.2)	< 0.001	0.458	18,899 (65.3)	19,021 (65.7)	0.286	0.009
HbA1c < 7%, n (%)	11,845 (35.7)	22,080 (44.2)	< 0.001	0.174	10,803 (37.3)	10,804 (37.3)	0.993	< 0.001
Creatinine, mg/dL	1.3 ± 5.7	1.3 ± 4.4	0.323	0.007	1.3 ± 5.8	1.2 ± 4.4	0.019	0.021

*PSM, propensity score matching; SMD, standardized mean difference; OAD, oral antidiabetic drugs; BMI, body mass index; HbA1c, glycated hemoglobin*

*Dual* = *SGLT-2 inhibitors* + *GLP-1 receptor agonists; SGLT-2* = *SGLT-2 inhibitors only (excluding GLP-1 receptor agonists)*

*Continuous variables are presented as mean* ± *SD; categorical variables are presented as n (%)*

### Primary outcome

**Fig. 2:**
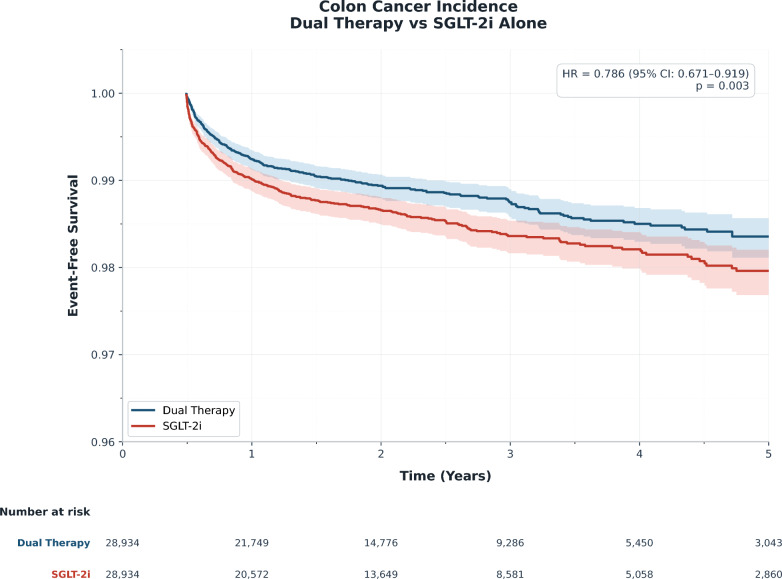
Kaplan–Meier curves for colon cancer-free survival: dual therapy vs SGLT-2 inhibitor monotherapy after propensity score matching

Colon cancer occurred in 285 patients (0.98%) in the dual therapy group and 344 patients (1.19%) in the SGLT-2 inhibitor only group, corresponding to incidence rates of 0.44 events per 100 person-years and 0.53 events per 100 person-years, respectively (eTable 2). Dual therapy was associated with a lower risk of colon cancer (HR 0.786, 95% CI 0.671–0.919; P = 0.003), representing a 21% relative risk reduction. The 5-year risk ratio was 0.828 (95% CI 0.709–0.968). The absolute risk difference was 0.21%, with a number needed to treat (NNT) of 490 over 5 years (Table 2). Kaplan–Meier curves demonstrated early and progressive separation, with 5-year cancer-free survival of 99.02% versus 98.81% (Fig. 2).

**Table 2:** Clinical Outcomes After Propensity Score Matching

Outcomes	Dual (SGLT-2i + GLP-1 RA)		SGLT-2i only		Risk Ratio	Hazard Ratio	Log-rank	E-value	NNT/NNH
	**N**	**Events**	**N**	**Events**	(95% CI)	(95% CI)	P value	(95% CI)	
**Primary outcome**									
Colon cancer	28,934	285	28,934	344	0.828 (0.709–0.968)	0.786 (0.671–0.919)	0.003	1.71 (1.22)	NNT = 490
**Secondary outcomes**									
Oncologic outcomes									
Colectomy	28,934	80	28,934	166	0.482 (0.368–0.631)	0.456 (0.264–0.790)	0.004	3.57 (2.55)	NNT = 336
Other gastrointestinal cancer	28,934	373	28,934	509	0.733 (0.642–0.837)	0.690 (0.604–0.789)	< 0.001	2.07 (1.68)	NNT = 213
Cardiovascular outcomes									
Major adverse cardiovascular events	28,934	3,025	28,934	2,881	1.050 (1.000–1.102)	0.990 (0.941–1.042)	0.693	1.28 (1.00)	NNH = 201
Heart failure	28,934	6,600	28,934	6,179	1.068 (1.036–1.101)	1.019 (0.985–1.055)	0.276	1.34 (1.23)	NNH = 69
Renal outcomes									
End-stage renal disease	28,934	612	28,934	718	0.852 (0.766–0.949)	0.808 (0.728–0.898)	< 0.001	1.63 (1.29)	NNT = 273
Major adverse kidney events	28,934	2,087	28,934	2,779	0.751 (0.711–0.793)	0.710 (0.667–0.755)	< 0.001	2.00 (1.84)	NNT = 42
Acute kidney injury	28,934	4,326	28,934	4,435	0.975 (0.938–1.014)	0.926 (0.884–0.971)	0.001	1.19 (1.00)	NNT = 265
Other outcomes									
All-cause mortality	28,934	1,077	28,934	1,526	0.706 (0.654–0.762)	0.658 (0.608–0.711)	< 0.001	2.18 (1.95)	NNT = 64
Acute pancreatitis	28,934	489	28,934	374	1.307 (1.143–1.494)	1.210 (1.145–1.275)	< 0.001	1.94 (1.55)	NNH = 252
Urinary tract infection	28,934	3,520	28,934	3,226	1.091 (1.043–1.141)	1.031 (0.983–1.081)	0.215	1.41 (1.25)	NNH = 98
Negative control outcomes									
Inguinal hernia	28,934	351	28,934	345	1.017 (0.878–1.179)	0.962 (0.840–1.102)	0.579	1.15 (1.00)	NNH = 4,822
Appendicitis	28,934	65	28,934	74	0.878 (0.632–1.221)	0.833 (0.593–1.169)	0.289	1.54 (1.00)	NNT = 3,215

CI, confidence interval; GLP-1, glucagon-like peptide-1; SGLT-2, sodium-glucose cotransporter-2

Risk ratio and hazard ratio are calculated with Dual group as reference (Dual vs. SGLT-2 inhibitor only)

### Co-Primary Analysis

The active comparator analysis comparing dual therapy versus GLP-1 RA monotherapy (N = 13,782 matched pairs) demonstrated that dual therapy was associated with lower colon cancer risk compared with GLP-1 RA monotherapy alone (115 events [0.8%] vs 179 events [1.3%]; HR 0.871, 95% CI 0.777–0.993; P = 0.041; eTable 12, Supplementary Materials), indicating that the combination confers additional benefit beyond GLP-1 RA alone.

### Secondary outcomes

Among oncologic outcomes, dual therapy was associated with lower risk of colectomy (HR 0.456, 95% CI 0.264–0.790; P = 0.004) and other gastrointestinal cancers (HR 0.690, 95% CI 0.604–0.789; P < 0.001), both below the Bonferroni-corrected threshold (α = 0.0045). No meaningful differences were observed in MACE (HR 0.990, P = 0.693) or heart failure (HR 1.019, P = 0.276). Renal outcomes were reduced: ESRD (HR 0.808, P < 0.001), MAKE (HR 0.710, P < 0.001), and acute kidney injury (HR 0.926, P = 0.001). All-cause mortality was markedly lower with dual therapy (HR 0.658, P < 0.001; NNT = 64).

**Fig. 3:**
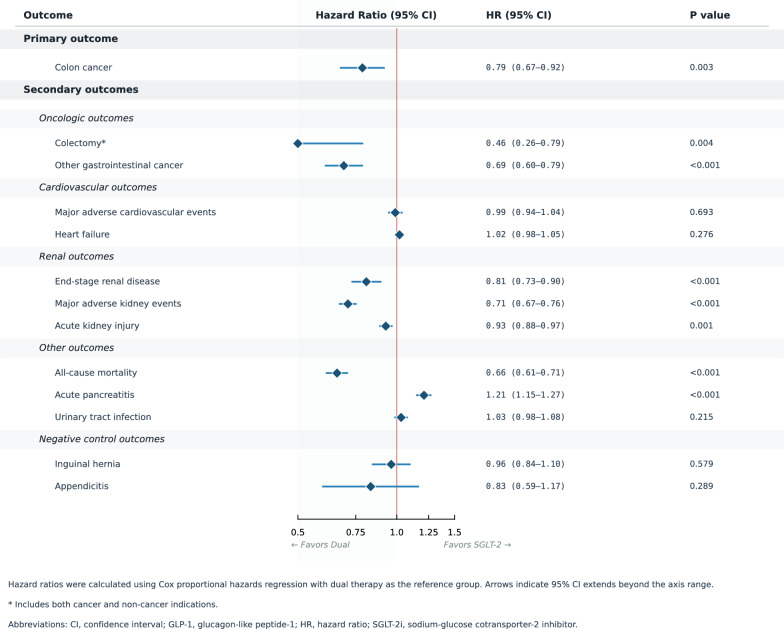
Forest plot of secondary clinical outcomes: dual therapy vs SGLT-2 inhibitor monotherapy

Acute pancreatitis was more frequent with dual therapy (HR 1.210, 95% CI 1.145–1.275; P < 0.001), consistent with the known GLP-1 RA class effect (NNH = 252). Negative control outcomes (inguinal hernia: HR 0.962, P = 0.579; appendicitis: HR 0.833, P = 0.289) showed no meaningful differences, supporting internal validity (Table 2, Fig. 3).

### Subgroup analyses

**Fig. 4:**
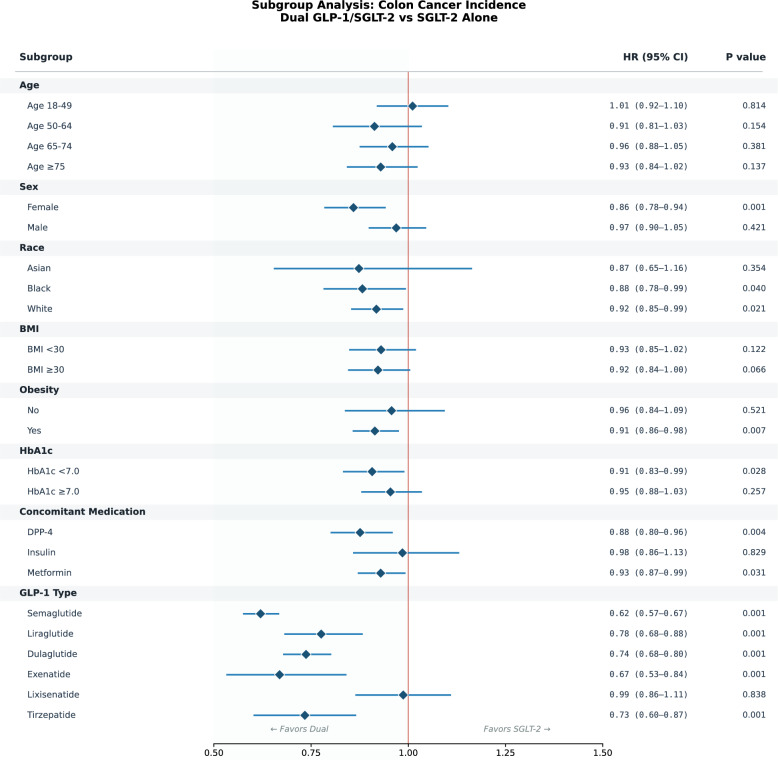
Subgroup analysis forest plot for the primary outcome (colon cancer incidence)

The protective association was broadly consistent across subgroups (Fig. 4, eTables 3–7). The association was stronger in females (HR 0.86, P = 0.001) than males (HR 0.97, P = 0.421; P-interaction = 0.042). Consistent associations were observed across racial groups: White (HR 0.92, P = 0.021), Black (HR 0.88, P = 0.040), and Asian (HR 0.87, P = 0.354). Among GLP-1 RA types, semaglutide-containing dual therapy showed the largest risk reduction (HR 0.62, 95% CI 0.57–0.67), followed by exenatide (HR 0.67), tirzepatide (HR 0.73), dulaglutide (HR 0.74), and liraglutide (HR 0.78). Lixisenatide showed no meaningful difference (HR 0.99, P = 0.838). The interaction by GLP-1 RA type was significant (P < 0.001).

### Sensitivity analyses

The primary finding was robust across all sensitivity analyses (eTable 2, Supplementary Materials). The 90-day landmark analysis (N = 27,456 per group) yielded consistent results (HR 0.77, 95% CI 0.65–0.90; P = 0.001). The 1-year landmark and 3-year follow-up showed consistent findings (HR 0.77, P = 0.006; HR 0.81, P = 0.028). Restriction to age ≥ 50 years (HR 0.78, P = 0.003) and US population only (HR 0.80, P = 0.012) demonstrated similar results.

The per-protocol analysis restricting to patients with ≥ 6 prescriptions of each agent (N = 5,752 per group) yielded consistent results (HR 0.823, 95% CI 0.638–0.972; P = 0.031), supporting that the association is not driven by transient concurrent use (eTable 11, Supplementary Materials).

Colonoscopy rates during follow-up were similar between groups (dual: 6,255 [21.6%] vs SGLT-2i: 6,086 [21.0%]; HR 1.038, 95% CI 0.996–1.082; P = 0.078; eTable 8b, Supplementary Materials), indicating that differential endoscopic surveillance is unlikely to explain the observed findings. Post-treatment improvements in BMI and HbA1c were greater with dual therapy, as illustrated in eFigure 2 (Supplementary Materials).

## Discussion

In this large target trial emulation study of post-polypectomy patients with T2DM, dual therapy with SGLT-2 inhibitor plus GLP-1 RA was associated with a 21% lower risk of colon cancer compared with SGLT-2 inhibitor monotherapy over 5-year follow-up. This association was accompanied by significant reductions in colectomy, other gastrointestinal cancers, renal outcomes, and all-cause mortality. The co-primary analysis demonstrated that dual therapy was also associated with lower colon cancer risk compared with GLP-1 RA monotherapy alone (HR 0.871; P = 0.041), indicating that the observed benefit is not attributable solely to the GLP-1 RA component but reflects additive or complementary effects of the combination. The null findings for negative control outcomes and comparable colonoscopy rates between groups support the internal validity of our study design.

To our knowledge, this is the first study to evaluate the association of dual SGLT-2 inhibitor plus GLP-1 RA therapy with colon cancer risk, and the first study to examine antidiabetic medications specifically in a post-polypectomy population. Prior studies examining individual agents reported SGLT-2 inhibitor monotherapy to be associated with a 20–29% reduction in CRC risk versus other antidiabetic agents [10–15], and GLP-1 RA monotherapy to be associated with a 23–30% reduction [18, 19]. A recent systematic review and meta-analysis further confirmed that GLP-1 RA use was associated with a 30% lower risk of colorectal cancer within 10 years of treatment initiation, alongside protective associations for multiple obesity-related malignancies including hepatocellular, pancreatic, and endometrial cancers.[31] Our co-primary analysis demonstrated that dual therapy conferred a further 13% reduction versus GLP-1 RA monotherapy alone (HR 0.871) suggests that the combination provides incremental oncologic benefit through complementary mechanistic pathways.

The mechanistic rationale for complementary effects is compelling. SGLT-2 inhibitors act through glucose deprivation and metabolic reprogramming of cancer cells, AMPK activation, ferroptosis induction, and suppression of the Wnt/β-catenin pathway [6–8, 32, 33]. GLP-1 RAs complement these effects by reducing systemic hyperinsulinemia — a key promoter of CRC in T2DM — lowering obesity-related inflammation, and exerting direct anti-proliferative effects on colonic epithelium [34, 35]. Simultaneously targeting multiple complementary oncogenic pathways may explain the incremental benefit beyond monotherapy with either agent. The greater improvements in BMI and HbA1c observed with dual therapy (eFigure 2) suggest that superior metabolic control may contribute mechanistically to the observed oncologic association.

Several notable subgroup findings deserve comment. The stronger association in females (HR 0.86) versus males (HR 0.97; P-interaction = 0.042) may reflect sex differences in colorectal cancer biology or differential metabolic response to GLP-1 RA therapy. Among GLP-1 RA types, semaglutide-containing dual therapy showed the strongest association (HR 0.62), potentially reflecting greater weight loss efficacy and more potent anti-inflammatory effects of semaglutide, consistent with its known superior efficacy for metabolic outcomes [36].

The significant reductions in renal outcomes align with evidence from cardiovascular outcome trials demonstrating additive cardiorenal benefits of combination therapy [21–23]. The 34% reduction in all-cause mortality (HR 0.658; NNT = 64) likely reflects the combined cardiovascular, renal, and potentially anticancer effects of dual therapy. The absence of a statistically meaningful cardiovascular benefit in this study is consistent with the established observation that MACE reduction is most pronounced in populations with existing cardiovascular disease, which was not specifically enriched for in our cohort.

Our study has several strengths. First, we employed a rigorous target trial emulation framework with explicit protocol specification, new-user design, and landmark analysis to address immortal time bias [24–27]. Second, we utilized a large global federated database with real-world data from 57 diverse healthcare organizations. Third, PSM achieved excellent covariate balance (SMD < 0.1 for all variables, including BMI [SMD 0.095]). Fourth, comprehensive sensitivity analyses were conducted, including a 90-day landmark analysis, a per-protocol analysis, a co-primary analysis comparing dual therapy against GLP-1 RA monotherapy, and empirical colonoscopy rate comparison. Fifth, 5-year follow-up allowed sufficient time for cancer detection. Sixth, E-value analysis (E-value 1.71; 95% CI lower bound 1.22) provides formal quantification of robustness to unmeasured confounding.

Several limitations should be acknowledged. First, as an observational study, causality cannot be established. Although tobacco use, alcohol use disorder, aspirin, NSAIDs, and family history were incorporated as PSM covariates and well balanced after matching, EHR-based capture rates for behavioral variables underestimate true prevalence, and residual confounding from unmeasured lifestyle factors cannot be fully excluded. The E-value of 1.71 indicates that an unmeasured confounder would need to be associated with both treatment and outcome by a relative risk of at least 1.71 to fully explain the observed association. In particular, advanced adenoma characteristics including villous histology, high-grade dysplasia, and large polyp size carry relative risks for subsequent colorectal cancer that may exceed the E-value threshold reported here, and differential distribution of advanced versus non-advanced adenomas between treatment groups cannot be excluded. Second, EHR data may have inaccuracies, such as misclassification. Our primary outcome was identified using ICD-10 diagnostic codes alone (C18.0-C18.9, C19, C20) without additional validation through manual chart review or pathology confirmation, which may result in outcome misclassification. While ICD-10-based algorithms have demonstrated good specificity for cancer diagnoses in large healthcare databases, potential misclassification could occur through miscoding of benign lesions as malignant or failure to capture cancers coded under alternative diagnostic categories. Such non-differential misclassification would likely bias estimates toward the null, making our findings conservative. Third, medication adherence could not be confirmed; our intent-to-treat design likely underestimates the true treatment effect, as non-adherence biases estimates toward the null. Fourth, we lacked data on polyp characteristics (size, histology, number, location) that influence cancer risk, as these pathology details are not consistently captured in structured EHR fields. Fifth, colectomy as an outcome includes procedures for non-cancer indications (e.g., diverticular disease, inflammatory bowel disease), reducing specificity; we were unable to restrict this to cancer-related colectomy given database constraints. Besides, we were unable to adjust for potential differences in colonoscopy surveillance frequency during follow-up, as patients may undergo procedures at facilities outside the TriNetX network; however, differential surveillance would bias toward the null, making our findings conservative Sixth, the combination therapy group has shorter maximum follow-up (common only after 2017). Seventh, the TriNetX platform does not support competing risk analyses (e.g., Fine-Gray models); however, given lower all-cause mortality in the dual therapy group (HR 0.658), competing risk from differential mortality would bias our primary estimate toward the null, rendering findings conservative. Eighth, time-varying exposure analysis is not supported; index-date medication classification cannot fully capture treatment switching or discontinuation. Ninth, we were unable to conduct a formal duration-response analysis due to limitations in the precision of cumulative exposure estimation from administrative prescription records. Future studies with detailed dispensing data or electronic medication administration records should address whether a biological gradient exists between cumulative SGLT-2i/GLP-1 RA exposure and CRC risk reduction.

## Conclusions

In this target trial emulation study of post-polypectomy patients with T2DM, dual therapy with SGLT-2 inhibitor plus GLP-1 RA was associated with a 21% lower risk of colon cancer compared with SGLT-2 inhibitor monotherapy. Co-primary analysis demonstrated that this association also persisted when comparing dual therapy against GLP-1 RA monotherapy alone (HR 0.871; P = 0.041), indicating additive or complementary chemopreventive effects of the combination. These findings support prospective investigation to confirm these associations. Given that combination therapy is already recommended for cardiometabolic indications, our findings provide additional rationale for this approach in post-polypectomy T2DM patients at elevated colorectal cancer risk.

## Supplementary Information

Additional file 1.

## Data Availability

No datasets were generated or analysed during the current study.

## Declarations

### Declaration of AI and AI-assisted technologies in the writing process

During the preparation of this work the author(s) used Claude by Anthropic in order to refine language and formatting. After using this tool, the authors reviewed and edited the content as needed and take full responsibility for the content of the publication.

## References

[1] Sung H, et al. Global Cancer Statistics 2020: GLOBOCAN estimates. CA Cancer J Clin. 2021;71:209–49. 33538338 10.3322/caac.21660

[2] Giovannucci E, et al. Diabetes and cancer: a consensus report. Diabetes Care. 2010;33:1674–85. 20587728 10.2337/dc10-0666 PMC2890380

[3] Vigneri P, et al. Diabetes and cancer. Endocr Relat Cancer. 2009;16:1103–23. 19620249 10.1677/ERC-09-0087

[4] Pollak M. Insulin and IGF signalling in neoplasia. Nat Rev Cancer. 2008;8:915–28. 19029956 10.1038/nrc2536

[5] Weinstein D, et al. Insulin analogues display IGF-I-like activities in cultured cancer cells. Diabetes Metab Res Rev. 2009;25:41–9. 19145584 10.1002/dmrr.912

[6] Sun M, et al. Unveiling the anticancer effects of SGLT-2i. Front Pharmacol. 2024;15:1369352. 38595915 10.3389/fphar.2024.1369352 PMC11002155

[7] Nakhaei A, et al. SGLT-2 inhibitors beyond diabetes: a new frontier in cancer treatment. Diabetes Res Clin Pract. 2025; 112925. 10.1016/j.diabres.2025.112925 41016479

[8] Villani LA, et al. Canagliflozin reduces cancer cell proliferation by inhibiting mitochondrial complex-I. Mol Metab. 2016;5:1048–56. 27689018 10.1016/j.molmet.2016.08.014 PMC5034684

[9] Fodde R, Smits R, Clevers H. APC, signal transduction and genetic instability in CRC. Nat Rev Cancer. 2001;1:55–67. 11900252 10.1038/35094067

[10] Tang H, et al. SGLT2 inhibitors and risk of cancer in T2DM: systematic review and meta-analysis. Diabetologia. 2017;60:1862–72. 28725912 10.1007/s00125-017-4370-8

[11] Chiang CH, et al. SGLT-2 inhibitors and outcome of patients with DM and CRC. J Gastroenterol Hepatol. 2024;39:902–7. 38296226 10.1111/jgh.16498

[12] Yang JY, et al. Colorectal cancer and SGLT-2 inhibitor use. Diabetologia. 2025;68:321.

[13] Wang JY, et al. SGLT-2 inhibitors and colorectal cancer risk. Diabetes Care. 2025;48:180–8.

[14] Lee J, et al. SGLT-2 inhibitors and colorectal cancer risk: a population-based cohort study. J Clin Endocrinol Metab. 2024 [in press]. 10.1210/clinem/dgae257 38661006

[15] Kim JH, et al. Sodium-glucose cotransporter 2 inhibitors and cancer incidence. JAMA Netw Open. 2023;6:e2314453.

[16] Drucker DJ. The cardiovascular biology of glucagon-like peptide-1. Cell Metab. 2016;24:15–30. 27345422 10.1016/j.cmet.2016.06.009

[17] Bendotti G, et al. Anti-inflammatory and immunological properties of GLP-1 RAs. Pharmacol Res. 2022;182:106320. 35738455 10.1016/j.phrs.2022.106320

[18] Wang L, et al. GLP-1 receptor agonists and colorectal cancer risk. JAMA Oncol. 2024;10:256–8. 38060218 10.1001/jamaoncol.2023.5573 PMC10704339

[19] Bushi G, et al. Association Between GLP1 RAs and Risk of Colorectal Cancer: systematic review and meta-analysis. Health Sci Rep. 2025;8:e70490. 39980820 10.1002/hsr2.70490 PMC11839483

[20] American Diabetes Association. Pharmacologic approaches to glycemic treatment: standards of care 2022. Diabetes Care. 2022;45:S125–43. 34964831 10.2337/dc22-S009

[21] Neuen BL, et al. Cardiovascular, kidney, and safety outcomes with GLP-1 RAs alone and in combination with SGLT2i. Circulation. 2024;150:1781–90. 39210781 10.1161/CIRCULATIONAHA.124.071689

[22] Scheen AJ. Cardiovascular and renal effects of GLP-1 RA and SGLT2i combination. Diabetes Metab. 2025;51:101594. 39608670 10.1016/j.diabet.2024.101594

[23] Apperloo EM, Neuen BL, et al. Efficacy and safety of SGLT2i with and without GLP-1 RA: SMART-C meta-analysis. Lancet Diabetes Endocrinol. 2024;12:545–57. 38991584 10.1016/S2213-8587(24)00155-4

[24] Hernan MA, Wang W, Leaf DE. Target trial emulation: a framework for causal inference. JAMA. 2022;328:2446–7. 36508210 10.1001/jama.2022.21383

[25] Hubbard RA, et al. Target trial emulation for observational studies. N Engl J Med. 2024;391:1975–7. 39588897 10.1056/NEJMp2407586

[26] Fu EL. Target trial emulation to improve causal inference. J Am Soc Nephrol. 2023;34:1305–14. 37131279 10.1681/ASN.0000000000000152 PMC10400102

[27] Simon-Tillaux N, et al. Observational analyses with the target trial emulation approach: systematic review. BMJ Open. 2024;14:e086595. 39532374 10.1136/bmjopen-2024-086595 PMC11574403

[28] Palchuk MB, et al. A global federated real-world data and analytics platform for research. JAMIA Open. 2023;6. 10.1093/jamiaopen/ooad035 PMC10182857 37193038

[29] Ludwig RJ, et al. Methodologies and applications of TriNetX. Front Pharmacol. 2025;16:1516126. 40129946 10.3389/fphar.2025.1516126 PMC11931024

[30] Ray WA. Evaluating medication effects outside clinical trials: new-user designs. Am J Epidemiol. 2003;158:915–20. 14585769 10.1093/aje/kwg231

[31] Ateiwi YA, Mahmood R, Wong HJ, Low CE, Yau CE, Lee ARYB, et al. Glucagon-like peptide-1 receptor agonists and the risk of obesity-related cancers: a systematic review and meta-analysis. Diabetes Res Clin Pract. 2026;234:113158. 41722869 10.1016/j.diabres.2026.113158

[32] Papadopoli D, et al. mTOR as a central regulator of lifespan and aging. F1000Res. 2019;8:F1000 Faculty Rev-998. 10.12688/f1000research.17196.1 PMC6611156 31316753

[33] Jiang X, Stockwell BR, Conrad M. Ferroptosis: mechanisms, biology and role in disease. Nat Rev Mol Cell Biol. 2021;22:266–82. 33495651 10.1038/s41580-020-00324-8 PMC8142022

[34] Lin A, et al. GLP-1 receptor agonists and cancer risk: mechanistic understanding and clinical evidence. Biomark Res. 2025;13:50. 40140925 10.1186/s40364-025-00765-3 PMC11948983

[35] Dai H, et al. GLP-1 receptor agonists and cancer risk in adults with obesity. JAMA Oncol. 2025;11:1186–93. 40839273 10.1001/jamaoncol.2025.2681 PMC12371547

[36] Wilding JPH, Batterham RL, Calanna S, Davies M, Van Gaal LF, Lingvay I, et al. Once-weekly semaglutide in adults with overweight or obesity. N Engl J Med. 2021;384:989–1002. 33567185 10.1056/NEJMoa2032183

