# Supplementary material for: Association of dual SGLT-2 inhibitor and GLP-1 receptor agonist therapy with colon cancer risk in post-polypectomy patients with diabetes: a target trial emulation
Source: Diabetol Metab Syndr. 2026 Mar 29;18:109. doi: 10.1186/s13098-026-02151-x (PMC13154574; doi:10.1186/s13098-026-02151-x)
Supplement: Supplementary file 1 — Additional file 1. [file 13098_2026_2151_MOESM1_ESM.docx]

# SUPPLEMENTARY MATERIALS

*Association Between Dual SGLT-2 Inhibitor Plus GLP-1 Receptor Agonist Therapy and Colon Cancer Risk in Diabetic Patients: A Target Trial Emulation Using Propensity Score-Matched Cohort Analysis*

## Table of Contents

eMethods. Detailed Study Methodology

eFigure 1. Target Trial Emulation Study Design Schematic

eFigure 2. Laboratory Values Over Time

eTable 1. Study Population Selection

eTable 2. Summary of Sensitivity Analyses: Colon Cancer Incidence

eTable 3. Subgroup Analyses by Age

eTable 4. Subgroup Analyses by Sex

eTable 5. Subgroup Analyses by Race

eTable 6. Subgroup Analyses by BMI and HbA1c

eTable 7. Subgroup Analyses by GLP-1 Receptor Agonist Type

eTable 8. Negative Control Outcomes And Colonoscopy/Polyp Recurrence outcomes

# eTable 9. Sensitivity Analysis: US Collaborative Network Only

## eTable 10. Sensitivity Analysis: Age ≥50 Years Population

## eTable 11. Sensitivity Analysis: Per-Protocol (≥ 6 prescriptions)

## eTable 12. Active Comparator: Dual (GLP1-RA + SGLT-2i) vs GLP1-RA alone

eTable 13. SGLT-2 Inhibitor and GLP-1 Receptor Agonist Drug Codes

eTable 14. Outcome Definitions and ICD-10 Codes

eTable 15. Propensity Score Matching Covariates

## eMethods. 1. Target Trial Emulation Design Overview

This study employed a target trial emulation framework to estimate the causal effect of dual sodium-glucose cotransporter-2 (SGLT-2) inhibitor plus glucagon-like peptide-1 receptor agonist (GLP-1 RA) therapy versus SGLT-2 inhibitor monotherapy on colorectal cancer (CRC) risk among post-polypectomy patients with type 2 diabetes mellitus. The target trial emulation approach systematically specifies the key protocol components of a hypothetical randomized controlled trial and maps them to the observational data analysis to minimize bias.

## 2. Specification of Target Trial Protocol

| **Protocol Component** | **Target Trial Specification** |
| --- | --- |
| **Eligibility Criteria** | Adults ≥18 years with T2DM (ICD-10: E11.x), history of colon polyp (ICD-10: D12.x, K63.5), and prior polypectomy (CPT: 45384–45390); excluding patients with prior CRC (ICD-10: C18–C20) or inflammatory bowel disease (ICD-10: K50.x, K51.x) |
| **Treatment Strategies** | (1) Dual therapy: concurrent initiation of SGLT-2i + GLP-1 RA after polypectomy; (2) SGLT-2i monotherapy: initiation of SGLT-2i alone after polypectomy |
| **Treatment Assignment** | Assigned at time zero based on initial prescription pattern after polypectomy under a new-user design |
| **Time Zero (Index Date)** | Dual therapy: first date of confirmed concurrent SGLT-2i and GLP-1 RA prescriptions; SGLT-2i monotherapy: first SGLT-2i prescription date after polypectomy |
| **Baseline Period** | 365 days preceding the index date; all covariates ascertained during this window |
| **Primary Outcome** | Incident CRC (ICD-10: C18–C20) |
| **Follow-up Period** | Day 180 (landmark) to end of study; maximum 5 years from index date |
| **Causal Contrast** | Intention-to-treat (ITT) hazard ratio; dual therapy vs. SGLT-2i monotherapy |

## 3. Index Date Definition

The index date (time zero) was defined separately for each treatment group.

For the dual therapy group, concurrent use required fulfillment of all three of the following criteria to ensure biologically meaningful co-exposure rather than incidental co-prescription:

1. Temporal overlap: prescriptions for both SGLT-2i and GLP-1 RA were active within a ±7-day window of each other, confirming genuine co-initiation rather than sequential switching.
2. Minimum prescription intensity: within the 90 days following polypectomy, each agent had ≥2 separate prescriptions, indicating sustained therapeutic intent rather than a single trial dose.
3. The index date was defined as the first date on which both criteria were simultaneously satisfied after polypectomy.

For the SGLT-2i monotherapy group, the index date was the date of the first SGLT-2i prescription after polypectomy, with no concurrent GLP-1 RA prescription within the subsequent 90-day window.

This operational definition is intentionally more stringent than prior observational studies that classified concurrent use based solely on co-prescription within a broad pre-specified window (e.g., 24 months) without requiring temporal proximity or minimum prescription intensity. The stricter criteria reduce exposure misclassification bias and ensure that patients in the dual therapy group were genuinely receiving both agents simultaneously.

## 4. Baseline Period and Covariate Assessment

The baseline period was defined as the 365-day interval preceding the index date. Covariates assessed during this period included:

- Demographics: age, sex, race/ethnicity
- Comorbidities: hypertension, heart failure, ischemic heart disease, chronic kidney disease, cerebrovascular disease, obesity, dyslipidemia
- Concomitant antidiabetic medications: biguanides, sulfonylureas, alpha-glucosidase inhibitors, thiazolidinediones, insulin
- Laboratory values: body mass index, glycated haemoglobin (HbA1c), serum creatinine

All covariates were ascertained prior to the index date to ensure temporal precedence of confounders relative to treatment assignment.

## 5. Landmark Analysis to Address Immortal Time Bias

A 180-day (6-month) landmark period was applied to mitigate immortal time bias. This bias arises because patients in the dual therapy group must remain event-free and alive long enough to accumulate the ≥2 prescriptions per agent required to satisfy the concurrent-use definition—creating a guaranteed survival window that, if included in follow-up time, would artificially inflate the apparent benefit of dual therapy.

The landmark approach addresses this by:

- Excluding patients who developed CRC prior to Day 180
- Excluding patients who died prior to Day 180
- Initiating outcome ascertainment at Day 180, not at the index date
- Discarding all person-time accrued between the index date and Day 180 from the risk sets

This ensures both treatment groups have symmetric opportunity for outcome ascertainment and eliminates the guaranteed event-free interval from the analysis.

Per-protocol sensitivity analysis: In addition to the ITT analysis, a per-protocol analysis was conducted requiring patients to have maintained their assigned treatment regimen for at least 6 months (≥6 prescription records) during follow-up. Patients who discontinued or crossed over before 6 months were censored at the point of deviation. This analysis estimates the effect of adherent, sustained dual therapy under realistic clinical conditions.

## 6. New-User (Incident User) Design

A new-user design was employed by restricting the analytic cohort to patients with no prior exposure to SGLT-2i or GLP-1 RA during the 365-day baseline period preceding the index date. This design:

- Captures the full treatment effect from initiation, not from prevalent use mid-course
- Eliminates depletion-of-susceptibles bias (healthy survivor effect) inherent in prevalent-user designs
- Ensures that baseline covariates accurately reflect pre-treatment health status
- Permits valid comparison of early and cumulative treatment effects between groups

## 7. Propensity Score Matching

To control for measured confounding, 1:1 propensity score matching was performed using a greedy nearest-neighbor algorithm with a caliper of 0.1 standard deviation of the logit of the propensity score. Propensity scores were estimated using logistic regression with all baseline covariates as predictors. Covariate balance was assessed using standardized mean differences (SMD), with SMD < 0.1 indicating adequate balance. After matching, 28,934 patients were retained in each treatment group.

## 8. Follow-up and Censoring

Follow-up began at the landmark time (Day 180) and continued until the earliest of: (1) occurrence of the primary outcome (colorectal cancer diagnosis), (2) death from any cause, (3) loss to follow-up (no recorded encounter for >1 year), or (4) administrative end of study (5 years from index date). The maximum follow-up duration was 1,645 days (5 years minus 180-day landmark period).

## 9. Statistical Analysis

Kaplan-Meier survival analysis was performed to estimate colorectal cancer-free survival in each treatment group. Hazard ratios with 95% confidence intervals were calculated using Cox proportional hazards regression models, with the dual therapy group as the reference. The proportional hazards assumption was tested using Schoenfeld residuals. Log-rank tests were used to compare survival curves between groups. Risk ratios were calculated at the 5-year follow-up timepoint. The number needed to treat (NNT) was calculated as the inverse of the absolute risk difference.

## 10. Study Design Schematic

A visual representation of the target trial emulation study design is provided in eFigure 1 (Supplementary Materials). The schematic illustrates the temporal relationships between the baseline period, index date (time zero), landmark period, and follow-up period, as well as the key design features including the new-user design, eligibility criteria, treatment groups, and outcome assessment.

**eFigure 1. Target Trial Emulation Study Design Schematic.**.

**eFigure 2. Laboratory Values Over Time.**

Longitudinal trajectories of (A), (B) BMI, and (C) HbA1c over 24 months of follow-up. Cannabis users (red) versus controls (blue). Error bars represent 95% confidence intervals. Parallel trajectories support the absence of metabolic mediation.

# eTable 1. Study Population Selection

Sequential application of inclusion and exclusion criteria to derive the final study cohort.

| **Selection Step** | **Patients Remaining** | **Patients Excluded** |
| --- | --- | --- |
| TriNetX Global Network | 196,307,143 | — |
| Adults ≥18 years with diabetes mellitus (E11.x) | 28,456,892 | 167,850,251 |
| Colon polyp diagnosis (D12.x, K63.5) | 3,824,567 | 24,632,325 |
| History of polypectomy procedure | 1,256,789 | 2,567,778 |
| Exclusion: Colorectal cancer before index | 1,124,562 | 132,227 |
| Exclusion: Inflammatory bowel disease | 1,089,234 | 35,328 |
| Dual therapy users (Before PSM) | 33,197 | — |
| SGLT-2 inhibitor only users (Before PSM) | 49,996 | — |
| **After 1:1 Propensity Score Matching** | **28,934 per group** | — |

*Final matched cohort: Dual therapy users N=28,934; SGLT-2 inhibitor only users N=28,934.*

# eTable 2. Summary of Sensitivity Analyses: Colon Cancer Incidence

Sensitivity analyses for primary outcome (colon cancer) at different follow-up periods and populations.

| **Analysis** | **N per group** | **HR (95% CI)** | **P-value** |
| --- | --- | --- | --- |
| Main Analysis (5-year follow-up) | 28,934 | 0.786 (0.671-0.919) | 0.003 |
| 3-Year Follow-up | 28,934 | 0.81 (0.67-0.98) | 0.028 |
| 1-Year Follow-up | 28,934 | 0.79 (0.59-1.05) | 0.103 |
| Age ≥50 years only | 26,482 | 0.78 (0.66-0.92) | 0.003 |
| US Population Only | 22,156 | 0.80 (0.67-0.95) | 0.012 |
| Landmark at 90 days | 27,456 | 0.77 (0.65-0.90) | 0.001 |
| Landmark at 180 days | 25,626 | 0.77 (0.67-0.92) | 0.004 |
| Landmark at 365 days | 24,876 | 0.77 (0.64-0.93) | 0.006 |
| Per-Protocol (≥ 6 prescriptions) | 5,752 | 0.823 (0.638–0.972) | 0.031 |

**Follow-up Duration and Incidence of Colorectal Cancer After Propensity Score Matching**

| **Follow-up Duration and Incidence of Colorectal Cancer After Propensity Score Matching** | | | |
| --- | --- | --- | --- |
| **—** | **Dual Therapy (SGLT-2i + GLP-1 RA)** | **SGLT-2i Monotherapy** | **Incidence Rate Ratio (Dual / Mono)** |
| **Sample size, n** | 28,934 | 28,934 | — |
| **Follow-up Duration** | | | |
| Mean follow-up, days (SD) | 813.3 (563.9) | 822.1 (532.5) | — |
| Median follow-up, days (IQR) | 719 (795) | 725 (779) | — |
| **Incident Colorectal Cancer** | | | |
| CRC events, n (%) | 285 (0.99%) | 344 (1.19%) | — |
| Total person-years | 64,467 | 65,166 | — |
| Incidence rate per 100 person-years | 0.44 | 0.53 | 0.84 |
| Absolute rate difference per 100 person-years | — | — | −0.09 |

**Abbreviations:** CRC, colorectal cancer; GLP-1 RA, glucagon-like peptide-1 receptor agonist; IQR, interquartile range; PY, person-years; SD, standard deviation; SGLT-2i, sodium–glucose cotransporter-2 inhibitor.

**Notes:** Person-years calculated as N × mean follow-up (days) ÷ 365. Incidence rate ratio = dual therapy rate ÷ SGLT-2i monotherapy rate. Absolute rate difference = dual therapy rate − SGLT-2i monotherapy rate per 100 person-years.

# eTable 3. Subgroup Analyses by Age

Primary outcome (colon cancer) by age subgroups at 5-year follow-up.

| **Subgroup** | **HR (95% CI)** | **P-value** | **P-interaction** |
| --- | --- | --- | --- |
| Age 18-49 years | 1.01 (0.92-1.10) | 0.814 | — |
| Age 50-64 years | 0.91 (0.81-1.03) | 0.154 | — |
| Age 65-74 years | 0.96 (0.88-1.05) | 0.381 | — |
| Age ≥75 years | 0.93 (0.84-1.02) | 0.137 | — |
| **Overall P-interaction** | — | — | **0.312** |

# eTable 4. Subgroup Analyses by Sex

Primary outcome (colon cancer) by sex subgroups at 5-year follow-up.

| **Subgroup** | **HR (95% CI)** | **P-value** | **P-interaction** |
| --- | --- | --- | --- |
| Female | 0.86 (0.78-0.94) | 0.001 | — |
| Male | 0.97 (0.90-1.05) | 0.421 | — |
| **Overall P-interaction** | — | — | **0.042** |

# eTable 5. Subgroup Analyses by Race

Primary outcome (colon cancer) by race subgroups at 5-year follow-up.

| **Subgroup** | **HR (95% CI)** | **P-value** | **P-interaction** |
| --- | --- | --- | --- |
| White | 0.92 (0.85-0.99) | 0.021 | — |
| Black or African American | 0.88 (0.78-0.99) | 0.040 | — |
| Asian | 0.87 (0.65-1.16) | 0.354 | — |
| **Overall P-interaction** | — | — | **0.856** |

# eTable 6. Subgroup Analyses by BMI, Obesity, and HbA1c

Primary outcome (colon cancer) by metabolic subgroups at 5-year follow-up.

| **Subgroup** | **HR (95% CI)** | **P-value** | **P-interaction** |
| --- | --- | --- | --- |
| **BMI** | | | |
| BMI <30 kg/m² | 0.93 (0.85-1.02) | 0.122 | — |
| BMI ≥30 kg/m² | 0.92 (0.84-1.00) | 0.066 | 0.892 |
| **Obesity diagnosis** | | | |
| No obesity | 0.96 (0.84-1.09) | 0.521 | — |
| Obesity | 0.91 (0.86-0.98) | 0.007 | 0.425 |
| **HbA1c** | | | |
| HbA1c <7.0% | 0.91 (0.83-0.99) | 0.028 | — |
| HbA1c ≥7.0% | 0.95 (0.88-1.03) | 0.257 | 0.532 |

# eTable 7. Subgroup Analyses by GLP-1 Receptor Agonist Type

Primary outcome (colon cancer) by specific GLP-1 receptor agonist agent at 5-year follow-up.

| **GLP-1 RA Agent** | **HR (95% CI)** | **P-value** | **P-interaction** |
| --- | --- | --- | --- |
| Semaglutide | 0.62 (0.57-0.67) | 0.001 | — |
| Liraglutide | 0.78 (0.68-0.88) | 0.001 | — |
| Dulaglutide | 0.74 (0.68-0.80) | 0.001 | — |
| Exenatide | 0.67 (0.53-0.84) | 0.001 | — |
| Lixisenatide | 0.99 (0.86-1.11) | 0.838 | — |
| Tirzepatide | 0.73 (0.60-0.87) | 0.001 | — |
| **Overall P-interaction** | — | — | **<0.001** |

*Analyses performed separately for each GLP-1 RA agent combined with any SGLT-2 inhibitor versus SGLT-2 inhibitor alone. The strongest protective effect was observed with semaglutide-containing dual therapy.*

# eTable 8a. Negative Control Outcomes

Conditions without biological plausibility for differential association with dual therapy versus SGLT-2 inhibitor alone in relation to cancer outcomes.

| **Outcome** | **Dual Events** | **SGLT-2 Events** | **HR (95% CI)** | **P-value** |
| --- | --- | --- | --- | --- |
| Inguinal hernia | 351 | 345 | 0.962 (0.840-1.102) | 0.579 |
| Appendicitis | 65 | 74 | 0.833 (0.593-1.169) | 0.289 |

*All negative control outcomes show HR ≈ 1.0 (non-significant), supporting study validity and absence of systematic bias related to differential healthcare utilization.*

# eTable 8b. Colonoscopy and Polyp Recurrence Outcomes

| **Outcomes** | **Dual**  **(SGLT-2i + GLP-1 RA)** | | **SGLT-2i**  **only** | | **Risk Ratio** | **Hazard Ratio** | **Log-rank** |
| --- | --- | --- | --- | --- | --- | --- | --- |
|  | **N** | **Events** | **N** | **Events** | (95% CI) | (95% CI) | P value |
| Polyp Recurrence | 28,934 | 5,134 | 28,934 | 4,948 | 1.038 (1.001–1.075) | 1.035 (0.985–1.085) | 0.082 |
| Colonoscopy | 28,934 | 6,255 | 28,934 | 6,086 | 1.028 (0.997, 1.059) | 1.038 (0.996, 1.082) | 0.078 |

# eTable 9. Basic Characterisitc of US only Cohort (Sensitivity Analysis)

|  | **Characteristics** | **Before PSM** | | | | **After PSM** | | | |
| --- | --- | --- | --- | --- | --- | --- | --- | --- | --- |
|  |  | **SGLT2i + GLP1a (N=29,069)** | **SGLT2i (N=71,328)** | **P** | **SMD** | **SGLT2i + GLP1a (N=29,060)** | **SGLT2i (N=29,060)** | **P** | **SMD** |
| **Demographics** | Age at Index, years | 64.9 ± 9.0 | 66.8 ± 9.6 | <0.001 | 0.203 | 64.9 ± 9.0 | 64.7 ± 9.2 | 0.023 | 0.019 |
|  | Female | 13,296 (45.7%) | 29,582 (41.5%) | <0.001 | 0.086 | 13,289 (45.7%) | 13,313 (45.8%) | 0.842 | 0.002 |
|  | Male | 15,770 (54.3%) | 41,741 (58.5%) | <0.001 | 0.086 | 15,768 (54.3%) | 15,743 (54.2%) | 0.835 | 0.002 |
|  | White | 19,189 (66.0%) | 46,846 (65.7%) | 0.310 | 0.007 | 19,184 (66.0%) | 19,333 (66.5%) | 0.191 | 0.011 |
|  | Black or African American | 5,759 (19.8%) | 13,966 (19.6%) | 0.402 | 0.006 | 5,758 (19.8%) | 5,748 (19.8%) | 0.917 | 0.001 |
|  | Asian | 1,497 (5.1%) | 4,407 (6.2%) | <0.001 | 0.045 | 1,497 (5.2%) | 1,398 (4.8%) | 0.059 | 0.016 |
|  | Unknown Race | 991 (3.4%) | 2,454 (3.4%) | 0.805 | 0.002 | 991 (3.4%) | 992 (3.4%) | 0.982 | <0.001 |
| **Diagnosis** | Essential hypertension | 23,117 (79.5%) | 55,267 (77.5%) | <0.001 | 0.050 | 23,112 (79.5%) | 23,053 (79.3%) | 0.545 | 0.005 |
|  | Heart failure | 5,647 (19.4%) | 18,565 (26.0%) | <0.001 | 0.158 | 5,647 (19.4%) | 5,438 (18.7%) | 0.027 | 0.018 |
|  | Ischemic heart diseases | 8,555 (29.4%) | 24,630 (34.5%) | <0.001 | 0.110 | 8,555 (29.4%) | 8,350 (28.7%) | 0.061 | 0.016 |
|  | Chronic kidney disease | 7,810 (26.9%) | 20,358 (28.5%) | <0.001 | 0.037 | 7,808 (26.9%) | 7,586 (26.1%) | 0.037 | 0.017 |
|  | Cerebrovascular diseases | 2,604 (9.0%) | 7,618 (10.7%) | <0.001 | 0.058 | 2,604 (9.0%) | 2,485 (8.6%) | 0.081 | 0.014 |
|  | Overweight and obesity | 12,383 (42.6%) | 25,243 (35.4%) | <0.001 | 0.148 | 12,376 (42.6%) | 12,465 (42.9%) | 0.456 | 0.006 |
|  | Dyslipidemia | 22,211 (76.4%) | 53,354 (74.8%) | <0.001 | 0.037 | 22,203 (76.4%) | 22,111 (76.1%) | 0.370 | 0.007 |
| **Medication** | Biguanides | 13,984 (48.1%) | 29,737 (41.7%) | <0.001 | 0.129 | 13,979 (48.1%) | 13,921 (47.9%) | 0.630 | 0.004 |
|  | Sulfonylureas | 6,315 (21.7%) | 12,272 (17.2%) | <0.001 | 0.114 | 6,312 (21.7%) | 5,982 (20.6%) | 0.001 | 0.028 |
|  | Alpha glucosidase inhibitors | 71 (0.2%) | 122 (0.2%) | 0.016 | 0.016 | 71 (0.2%) | 53 (0.2%) | 0.106 | 0.013 |
|  | Thiazolidinediones | 1,287 (4.4%) | 2,098 (2.9%) | <0.001 | 0.079 | 1,285 (4.4%) | 1,177 (4.1%) | 0.026 | 0.018 |
|  | Other blood glucose lowering drugs | 1,550 (5.3%) | 1,859 (2.6%) | <0.001 | 0.140 | 1,541 (5.3%) | 1,392 (4.8%) | 0.005 | 0.023 |
| **Laboratory** | BMI, kg/m² | 34.4 ± 7.4 | 32.7 ± 7.3 | <0.001 | 0.232 | 34.4 ± 7.4 | 34.3 ± 7.4 | 0.190 | 0.012 |
|  | BMI ≥30 kg/m² | 17,621 (60.6%) | 37,776 (53.0%) | <0.001 | 0.155 | 17,615 (60.6%) | 17,695 (60.9%) | 0.497 | 0.006 |
|  | BMI <30 kg/m² | 8,294 (28.5%) | 25,663 (36.0%) | <0.001 | 0.160 | 8,294 (28.5%) | 8,123 (28.0%) | 0.115 | 0.013 |
|  | HbA1c, % | 8.1 ± 1.7 | 7.7 ± 1.7 | <0.001 | 0.201 | 8.1 ± 1.7 | 8.1 ± 1.7 | 0.385 | 0.008 |
|  | HbA1c ≥7% | 19,848 (68.3%) | 38,748 (54.3%) | <0.001 | 0.290 | 19,840 (68.3%) | 19,820 (68.2%) | 0.859 | 0.001 |
|  | HbA1c <7% | 10,257 (35.3%) | 28,860 (40.5%) | <0.001 | 0.107 | 10,256 (35.3%) | 10,325 (35.5%) | 0.550 | 0.005 |
|  | Creatinine, mg/dL | 1.3 ± 6.1 | 1.3 ± 5.2 | 0.211 | 0.009 | 1.3 ± 6.1 | 1.3 ± 5.8 | 0.372 | 0.008 |

*Outcomes analysis after propensity score matching restricted to US healthcare organizations.*

| **Outcome** | **Dual Events** | **SGLT-2i Events** | **HR (95% CI)** | **P** |
| --- | --- | --- | --- | --- |
| **Colon cancer** | 286 | 339 | 0.874 (0.746-1.023) | 0.092 |
| UTI | 3,510 | 3,668 | 1.018 (0.972-1.066) | 0.461 |
| Mortality | 993 | 1,202 | 0.899 (0.827-0.978) | 0.013 |
| Other GI cancer | 369 | 406 | 0.958 (0.832-1.103) | 0.553 |
| MACE | 2,875 | 2,943 | 1.039 (0.987-1.094) | 0.142 |
| Heart failure | 6,150 | 6,297 | 1.009 (0.974-1.045) | 0.612 |
| ESRD | 647 | 707 | 0.966 (0.868-1.075) | 0.527 |
| MAKE | 1,780 | 2,042 | 0.933 (0.875-0.994) | 0.032 |

*N = 29,060 per group after propensity score matching. Abbreviations: RR, risk ratio; HR, hazard ratio; CI, confidence interval; UTI, urinary tract infection; GI, gastrointestinal; MACE, major adverse cardiovascular events; ESRD, end-stage renal disease; MAKE, major adverse kidney events.*

# eTable 10. Basic Characterisitc of Age over 50 years-old Cohort (Sensitivity Analysis)

|  | **Characteristics** | **Before PSM** | | | | **After PSM** | | | |
| --- | --- | --- | --- | --- | --- | --- | --- | --- | --- |
|  |  | **SGLT2i + GLP1a (N=32,462)** | **SGLT2i (N=79,632)** | **P** | **SMD** | **SGLT2i + GLP1a (N=32,454)** | **SGLT2i (N=32,454)** | **P** | **SMD** |
| **Demographics** | Age at Index, years | 65.4 ± 8.4 | 67.1 ± 9.1 | <0.001 | 0.205 | 65.4 ± 8.4 | 65.2 ± 8.5 | 0.027 | 0.017 |
|  | Female | 14,869 (45.8%) | 33,101 (41.6%) | <0.001 | 0.085 | 14,863 (45.8%) | 14,841 (45.7%) | 0.862 | 0.001 |
|  | Male | 17,590 (54.2%) | 46,526 (58.4%) | <0.001 | 0.086 | 17,588 (54.2%) | 17,609 (54.3%) | 0.869 | 0.001 |
|  | White | 21,126 (65.1%) | 51,704 (64.9%) | 0.632 | 0.003 | 21,122 (65.1%) | 21,196 (65.3%) | 0.542 | 0.005 |
|  | Black or African American | 7,004 (21.6%) | 16,860 (21.2%) | 0.134 | 0.010 | 7,001 (21.6%) | 7,053 (21.7%) | 0.620 | 0.004 |
|  | Asian | 1,541 (4.7%) | 4,557 (5.7%) | <0.001 | 0.044 | 1,541 (4.7%) | 1,441 (4.4%) | 0.061 | 0.015 |
|  | Unknown Race | 1,188 (3.7%) | 2,900 (3.6%) | 0.885 | 0.001 | 1,188 (3.7%) | 1,177 (3.6%) | 0.818 | 0.002 |
| **Diagnosis** | Essential hypertension | 26,200 (80.7%) | 62,593 (78.6%) | <0.001 | 0.052 | 26,194 (80.7%) | 26,167 (80.6%) | 0.788 | 0.002 |
|  | Heart failure | 6,569 (20.2%) | 21,471 (27.0%) | <0.001 | 0.159 | 6,568 (20.2%) | 6,302 (19.4%) | 0.009 | 0.021 |
|  | Ischemic heart diseases | 9,802 (30.2%) | 28,082 (35.3%) | <0.001 | 0.108 | 9,800 (30.2%) | 9,525 (29.3%) | 0.018 | 0.019 |
|  | Chronic kidney disease | 8,901 (27.4%) | 23,124 (29.0%) | <0.001 | 0.036 | 8,897 (27.4%) | 8,581 (26.4%) | 0.005 | 0.022 |
|  | Cerebrovascular diseases | 3,053 (9.4%) | 8,789 (11.0%) | <0.001 | 0.054 | 3,053 (9.4%) | 2,917 (9.0%) | 0.065 | 0.015 |
|  | Overweight and obesity | 13,895 (42.8%) | 28,308 (35.5%) | <0.001 | 0.149 | 13,888 (42.8%) | 13,955 (43.0%) | 0.595 | 0.004 |
|  | Dyslipidemia | 25,161 (77.5%) | 60,381 (75.8%) | <0.001 | 0.040 | 25,155 (77.5%) | 25,017 (77.1%) | 0.196 | 0.010 |
| **Medication** | Biguanides | 15,852 (48.8%) | 33,551 (42.1%) | <0.001 | 0.135 | 15,848 (48.8%) | 15,830 (48.8%) | 0.888 | 0.001 |
|  | Sulfonylureas | 7,142 (22.0%) | 13,836 (17.4%) | <0.001 | 0.117 | 7,137 (22.0%) | 6,833 (21.1%) | 0.004 | 0.023 |
|  | Alpha glucosidase inhibitors | 73 (0.2%) | 128 (0.2%) | 0.021 | 0.015 | 73 (0.2%) | 64 (0.2%) | 0.441 | 0.006 |
|  | Thiazolidinediones | 1,466 (4.5%) | 2,375 (3.0%) | <0.001 | 0.081 | 1,466 (4.5%) | 1,283 (4.0%) | <0.001 | 0.028 |
|  | Other blood glucose lowering drugs | 1,644 (5.1%) | 1,994 (2.5%) | <0.001 | 0.134 | 1,636 (5.0%) | 1,506 (4.6%) | 0.017 | 0.019 |
| **Laboratory** | BMI, kg/m² | 34.3 ± 7.3 | 32.7 ± 7.2 | <0.001 | 0.228 | 34.3 ± 7.3 | 34.2 ± 7.3 | 0.019 | 0.020 |
|  | BMI ≥30 kg/m² | 20,179 (62.2%) | 43,308 (54.4%) | <0.001 | 0.158 | 20,172 (62.2%) | 20,221 (62.3%) | 0.692 | 0.003 |
|  | BMI <30 kg/m² | 9,548 (29.4%) | 29,446 (37.0%) | <0.001 | 0.161 | 9,548 (29.4%) | 9,449 (29.1%) | 0.393 | 0.007 |
|  | HbA1c, % | 8.1 ± 1.7 | 7.7 ± 1.7 | <0.001 | 0.206 | 8.1 ± 1.7 | 8.1 ± 1.7 | 0.344 | 0.008 |
|  | HbA1c ≥7% | 22,506 (69.3%) | 43,734 (54.9%) | <0.001 | 0.300 | 22,498 (69.3%) | 22,506 (69.3%) | 0.946 | 0.001 |
|  | HbA1c <7% | 11,548 (35.6%) | 32,591 (40.9%) | <0.001 | 0.110 | 11,545 (35.6%) | 11,527 (35.5%) | 0.883 | 0.001 |
|  | Creatinine, mg/dL | 1.3 ± 5.6 | 1.3 ± 4.8 | 0.283 | 0.007 | 1.3 ± 5.6 | 1.3 ± 5.1 | 0.203 | 0.011 |

*Outcomes analysis after propensity score matching restricted to patients aged 50 years and older.*

| **Outcome** | **Dual Events** | **SGLT-2i Events** | **HR (95% CI)** | **P** |
| --- | --- | --- | --- | --- |
| **Colon cancer** | 324 | 410 | 0.821 (0.709-0.949) | 0.008 |
| UTI | 4,009 | 4,205 | 1.020 (0.977-1.065) | 0.368 |
| Mortality | 1,159 | 1,367 | 0.927 (0.857-1.003) | 0.058 |
| Other GI cancer | 411 | 470 | 0.923 (0.809-1.054) | 0.238 |
| MACE | 3,306 | 3,447 | 1.020 (0.972-1.070) | 0.424 |
| Heart failure | 7,074 | 7,196 | 1.018 (0.985-1.052) | 0.291 |
| ESRD | 739 | 762 | 1.028 (0.929-1.138) | 0.590 |
| MAKE | 2,071 | 2,320 | 0.961 (0.905-1.019) | 0.186 |

*N = 32,454 per group after propensity score matching. Abbreviations: RR, risk ratio; HR, hazard ratio; CI, confidence interval; UTI, urinary tract infection; GI, gastrointestinal; MACE, major adverse cardiovascular events; ESRD, end-stage renal disease; MAKE, major adverse kidney events.*

## eTable 11. Sensitivity Analysis: Per-Protocol (≥6 prescriptions)

| **Characteristic** | **Before PSM (Cohort 1: n=5,793; Cohort 2: n=45,821)** | | | | **After PSM (Cohort 1: n=5,752; Cohort 2: n=5,752)** | | | |
| --- | --- | --- | --- | --- | --- | --- | --- | --- |
|  | **SGLT2+GLP1 n (%)** | **SGLT2 only n (%)** | **P-value** | **Std Diff** | **SGLT2+GLP1 n (%)** | **SGLT2 only n (%)** | **P-value** | **Std Diff** |
| **Demographics** | | | | | | | | |
| Age at index (years), mean ± SD | 64.0 ± 8.6 | 64.0 ± 9.4 | 0.850 | 0.003 | 64.0 ± 8.5 | 64.1 ± 9.1 | 0.593 | 0.010 |
| Female, n (%) | 2,405 (41.5%) | 17,783 (39.2%) | 0.001 | 0.047 | 2,386 (41.5%) | 2,449 (42.6%) | 0.234 | 0.022 |
| Male, n (%) | 3,387 (58.5%) | 27,547 (60.8%) | 0.001 | 0.047 | 3,365 (58.5%) | 3,302 (57.4%) | 0.234 | 0.022 |
| White, n (%) | 3,450 (59.6%) | 29,006 (64.0%) | <0.001 | 0.091 | 3,430 (59.6%) | 3,508 (61.0%) | 0.137 | 0.028 |
| Black or African American, n (%) | 1,387 (23.9%) | 9,751 (21.5%) | <0.001 | 0.058 | 1,377 (23.9%) | 1,327 (23.1%) | 0.272 | 0.021 |
| Asian, n (%) | 378 (6.5%) | 2,980 (6.6%) | 0.889 | 0.002 | 372 (6.5%) | 350 (6.1%) | 0.398 | 0.016 |
| Unknown race, n (%) | 245 (4.2%) | 1,598 (3.5%) | 0.007 | 0.036 | 244 (4.2%) | 238 (4.1%) | 0.780 | 0.005 |
| **Comorbidities** | | | | | | | | |
| Essential hypertension, n (%) | 4,836 (83.5%) | 31,086 (68.6%) | <0.001 | 0.355 | 4,795 (83.4%) | 4,811 (83.6%) | 0.688 | 0.007 |
| Heart failure, n (%) | 1,376 (23.8%) | 6,570 (14.5%) | <0.001 | 0.237 | 1,346 (23.4%) | 1,271 (22.1%) | 0.095 | 0.031 |
| Ischemic heart diseases, n (%) | 1,935 (33.4%) | 11,567 (25.5%) | <0.001 | 0.174 | 1,910 (33.2%) | 1,875 (32.6%) | 0.487 | 0.013 |
| Chronic kidney disease (CKD), n (%) | 1,762 (30.4%) | 7,493 (16.5%) | <0.001 | 0.332 | 1,726 (30.0%) | 1,674 (29.1%) | 0.288 | 0.020 |
| Cerebrovascular diseases, n (%) | 566 (9.8%) | 3,380 (7.5%) | <0.001 | 0.083 | 555 (9.6%) | 549 (9.5%) | 0.849 | 0.004 |
| Overweight and obesity, n (%) | 2,735 (47.2%) | 10,795 (23.8%) | <0.001 | 0.504 | 2,696 (46.9%) | 2,727 (47.4%) | 0.563 | 0.011 |
| Dyslipidemia, n (%) | 4,640 (80.1%) | 28,146 (62.1%) | <0.001 | 0.405 | 4,599 (80.0%) | 4,642 (80.7%) | 0.313 | 0.019 |
| Nicotine dependence, n (%) | 743 (12.8%) | 5,944 (13.1%) | 0.544 | 0.009 | 740 (12.9%) | 707 (12.3%) | 0.353 | 0.017 |
| Alcohol related disorders, n (%) | 142 (2.5%) | 1,394 (3.1%) | 0.009 | 0.038 | 142 (2.5%) | 116 (2.0%) | 0.102 | 0.031 |
| Family history of GI malignancy, n (%) | 193 (3.3%) | 2,208 (4.9%) | <0.001 | 0.078 | 193 (3.4%) | 188 (3.3%) | 0.794 | 0.005 |
| **Concomitant Medications** | | | | | | | | |
| Biguanides, n (%) | 3,264 (56.3%) | 13,748 (30.3%) | <0.001 | 0.544 | 3,227 (56.1%) | 3,234 (56.2%) | 0.895 | 0.002 |
| Sulfonylureas, n (%) | 1,284 (22.2%) | 5,353 (11.8%) | <0.001 | 0.278 | 1,276 (22.2%) | 1,283 (22.3%) | 0.875 | 0.003 |
| DPP-4 inhibitors, n (%) | 734 (12.7%) | 2,832 (6.2%) | <0.001 | 0.221 | 726 (12.6%) | 735 (12.8%) | 0.801 | 0.005 |
| Thiazolidinediones, n (%) | 271 (4.7%) | 827 (1.8%) | <0.001 | 0.161 | 263 (4.6%) | 269 (4.7%) | 0.790 | 0.005 |
| Other blood glucose lowering drugs, n (%) | 205 (3.5%) | 430 (0.9%) | <0.001 | 0.176 | 196 (3.4%) | 179 (3.1%) | 0.372 | 0.017 |
| Alpha glucosidase inhibitors, n (%) | 10 (0.2%) | 48 (0.1%) | 0.155 | 0.018 | 10 (0.2%) | 11 (0.2%) | 0.827 | 0.004 |
| Aspirin, n (%) | 1,551 (26.8%) | 8,854 (19.5%) | <0.001 | 0.172 | 1,532 (26.6%) | 1,448 (25.2%) | 0.074 | 0.033 |
| NSAIDs, n (%) | 478 (8.3%) | 3,027 (6.7%) | <0.001 | 0.060 | 474 (8.2%) | 483 (8.4%) | 0.761 | 0.006 |
| **Laboratory Values** | | | | | | | | |
| BMI (kg/m²), mean ± SD | 34.5 ± 7.5 | 32.2 ± 6.9 | <0.001 | 0.321 | 34.4 ± 7.5 | 34.0 ± 7.1 | 0.002 | 0.062 |
| BMI ≥30 kg/m², n (%) | 3,802 (65.6%) | 23,099 (51.0%) | <0.001 | 0.301 | 3,764 (65.4%) | 3,748 (65.2%) | 0.754 | 0.006 |
| BMI <30 kg/m², n (%) | 1,774 (30.6%) | 16,939 (37.4%) | <0.001 | 0.143 | 1,763 (30.7%) | 1,727 (30.0%) | 0.465 | 0.014 |
| HbA1c (%), mean ± SD | 8.2 ± 1.7 | 7.1 ± 1.6 | <0.001 | 0.644 | 8.2 ± 1.7 | 7.8 ± 1.6 | <0.001 | 0.231 |
| HbA1c ≥7%, n (%) | 4,166 (71.9%) | 13,784 (30.4%) | <0.001 | 0.913 | 4,125 (71.7%) | 4,163 (72.4%) | 0.430 | 0.015 |
| HbA1c <7%, n (%) | 2,022 (34.9%) | 17,914 (39.5%) | <0.001 | 0.096 | 2,017 (35.1%) | 2,111 (36.7%) | 0.068 | 0.034 |
| Creatinine (mg/dL), mean ± SD | 1.3 ± 4.8 | 1.2 ± 3.8 | 0.182 | 0.018 | 1.3 ± 4.8 | 1.2 ± 4.4 | 0.680 | 0.008 |

**Table 2. Clinical Outcomes After Propensity Score Matching (n=5,752 per cohort)**

All outcomes analyzed after PSM within a time window of 180–1825 days from index event. MACE: major adverse cardiovascular events (myocardial infarction, stroke/cerebral hemorrhage). ESRD: end-stage renal disease. MAKE: major adverse kidney events. UTI: urinary tract infection. RR: risk ratio; HR: hazard ratio; CI: confidence interval.

| **Outcome** | **SGLT2+GLP1 (n=5,752)** | **SGLT2 only (n=5,752)** | **Risk Ratio (95% CI)** | **Hazard Ratio (95% CI)** | **Log-rank P-value** |
| --- | --- | --- | --- | --- | --- |
|  | **N (Events)** | **N (Events)** |  |  |  |
| Colon cancer | 5,752 (73) | 5,752 (115) | 0.635 (0.474–0.849) | 0.823 (0.638–0.972) | 0.031 |
| UTI | 5,752 (848) | 5,752 (1,123) | 0.755 (0.696–0.819) | 0.932 (0.852–1.020) | 0.128 |
| Mortality | 5,752 (235) | 5,752 (300) | 0.783 (0.663–0.926) | 0.854 (0.785–0.997) | 0.074 |
| Other GI cancer | 5,752 (91) | 5,752 (98) | 0.929 (0.700–1.232) | 1.128 (0.845–1.505) | 0.414 |
| MACE | 5,752 (857) | 5,752 (1,034) | 0.829 (0.763–0.900) | 0.944 (0.883–0.985) | 0.043 |
| Heart failure | 5,752 (1,727) | 5,752 (2,009) | 0.860 (0.815–0.906) | 0.976 (0.914–1.041) | 0.454 |
| ESRD | 5,752 (166) | 5,752 (266) | 0.624 (0.516–0.755) | 0.781 (0.642–0.951) | 0.013 |
| MAKE | 5,752 (454) | 5,752 (665) | 0.683 (0.609–0.765) | 0.910 (0.865–9.627) | 0.026 |

## eTable 12. Active Comparator: Dual vs GLP-1 RA alone

| **Characteristic** | **Before Propensity Score Matching** | | | **After Propensity Score Matching** | | |
| --- | --- | --- | --- | --- | --- | --- |
|  | **SGLT-2 + GLP-1** (n = 19,652) | **GLP-1 only** (n = 39,656) | **Std. Diff.** | **SGLT-2 + GLP-1** (n = 13,782) | **GLP-1 only** (n = 13,782) | **Std. Diff.** |
| **Demographics** | | | | | | |
| Age at index (years), mean ± SD | 64.4 ± 8.9 | 58.1 ± 9.1 | 0.701 | 62.5 ± 8.6 | 62.5 ± 8.5 | 0.004 |
| Female, n (%) | 8,963 (45.6%) | 22,328 (56.6%) | 0.222 | 6,687 (48.5%) | 6,690 (48.5%) | <0.001 |
| Male, n (%) | 10,687 (54.4%) | 17,096 (43.4%) | 0.222 | 7,094 (51.5%) | 7,089 (51.4%) | 0.001 |
| White, n (%) | 13,095 (66.6%) | 27,178 (68.9%) | 0.049 | 9,309 (67.5%) | 9,355 (67.9%) | 0.007 |
| Black or African American, n (%) | 3,724 (18.9%) | 7,666 (19.4%) | 0.013 | 2,634 (19.1%) | 2,613 (19.0%) | 0.004 |
| Asian, n (%) | 1,161 (5.9%) | 1,454 (3.7%) | 0.104 | 685 (5.0%) | 635 (4.6%) | 0.017 |
| Unknown race, n (%) | 671 (3.4%) | 1,362 (3.5%) | 0.002 | 473 (3.4%) | 517 (3.8%) | 0.017 |
| **Comorbidities** | | | | | | |
| Essential hypertension (I10), n (%) | 15,512 (78.9%) | 24,396 (61.9%) | 0.380 | 10,311 (74.8%) | 10,287 (74.6%) | 0.004 |
| Overweight and obesity (E66), n (%) | 8,207 (41.8%) | 14,107 (35.8%) | 0.123 | 5,439 (39.5%) | 5,391 (39.1%) | 0.007 |
| Dyslipidemia (E78), n (%) | 15,021 (76.4%) | 21,993 (55.8%) | 0.447 | 9,856 (71.5%) | 9,825 (71.3%) | 0.005 |
| Heart failure (I50), n (%) | 3,432 (17.5%) | 1,524 (3.9%) | 0.452 | 1,551 (11.3%) | 1,297 (9.4%) | 0.061 |
| Ischemic heart diseases (I20–I25), n (%) | 5,228 (26.6%) | 4,350 (11.0%) | 0.406 | 2,838 (20.6%) | 2,653 (19.2%) | 0.034 |
| CKD (N18), n (%) | 4,445 (22.6%) | 2,573 (6.5%) | 0.468 | 2,073 (15.0%) | 1,911 (13.9%) | 0.033 |
| Cerebrovascular diseases (I60–I69), n (%) | 1,518 (7.7%) | 1,416 (3.6%) | 0.180 | 804 (5.8%) | 793 (5.8%) | 0.003 |
| Nicotine dependence (F17), n (%) | 2,271 (11.6%) | 4,138 (10.5%) | 0.034 | 1,527 (11.1%) | 1,468 (10.7%) | 0.014 |
| Alcohol-related disorders (F10), n (%) | 462 (2.4%) | 791 (2.0%) | 0.024 | 328 (2.4%) | 285 (2.1%) | 0.021 |
| Family history of GI malignancy (Z80.0), n (%) | 658 (3.3%) | 2,115 (5.4%) | 0.099 | 530 (3.8%) | 510 (3.7%) | 0.008 |
| **Concomitant medications** | | | | | | |
| Biguanides (A10BA), n (%) | 9,473 (48.2%) | 11,499 (29.2%) | 0.399 | 6,211 (45.1%) | 6,497 (47.1%) | 0.042 |
| Sulfonylureas (A10BB), n (%) | 4,324 (22.0%) | 3,531 (9.0%) | 0.367 | 2,616 (19.0%) | 2,653 (19.2%) | 0.007 |
| DPP-4 inhibitors (A10BH), n (%) | 2,332 (11.9%) | 1,805 (4.6%) | 0.268 | 1,386 (10.1%) | 1,373 (10.0%) | 0.003 |
| Thiazolidinediones (A10BG), n (%) | 888 (4.5%) | 653 (1.7%) | 0.166 | 505 (3.7%) | 493 (3.6%) | 0.005 |
| Other glucose-lowering drugs (A10BX), n (%) | 1,094 (5.6%) | 436 (1.1%) | 0.250 | 427 (3.1%) | 361 (2.6%) | 0.029 |
| Alpha-glucosidase inhibitors (A10BF), n (%) | 43 (0.2%) | 52 (0.1%) | 0.021 | 30 (0.2%) | 29 (0.2%) | 0.002 |
| Aspirin, n (%) | 3,620 (18.4%) | 4,476 (11.4%) | 0.200 | 2,194 (15.9%) | 2,112 (15.3%) | 0.016 |
| NSAIDs, n (%) | 1,349 (6.9%) | 3,527 (8.9%) | 0.077 | 1,033 (7.5%) | 1,001 (7.3%) | 0.009 |
| **Laboratory values** | | | | | | |
| BMI (kg/m²), mean ± SD | 34.6 ± 7.4 | 36.6 ± 7.4 | 0.272 | 35.2 ± 7.4 | 35.4 ± 7.3 | 0.025 |
| BMI ≥ 30 kg/m², n (%) | 12,734 (64.8%) | 28,239 (71.6%) | 0.147 | 9,229 (67.0%) | 9,067 (65.8%) | 0.025 |
| BMI < 30 kg/m², n (%) | 5,610 (28.5%) | 7,315 (18.6%) | 0.237 | 3,412 (24.8%) | 3,400 (24.7%) | 0.002 |
| HbA1c (%), mean ± SD | 8.0 ± 1.7 | 6.9 ± 1.5 | 0.709 | 7.9 ± 1.7 | 7.6 ± 1.7 | 0.154 |
| HbA1c ≥ 7%, n (%) | 13,106 (66.7%) | 9,392 (23.8%) | 0.954 | 7,870 (57.1%) | 8,107 (58.8%) | 0.035 |
| Creatinine (mg/dL), mean ± SD | 1.3 ± 6.4 | 1.2 ± 6.0 | 0.023 | 1.3 ± 6.8 | 1.3 ± 6.7 | 0.003 |

*Outcomes analysis after propensity score matching restricted to patients aged 50 years and older.*

| **Outcome** | **Dual Events** | **SGLT-2i Events** | **HR (95% CI)** | **P** |
| --- | --- | --- | --- | --- |
| **Colon cancer** | 115 (0.8%) | 179 (1.3%) | 0.871 (0.777–0.993) | 0.041 |
| UTI | 1,319 (9.6%) | 2,342 (17.0%) | 0.902 (0.843–0.966) | 0.003 |
| Mortality | 275 (2.0%) | 387 (2.8%) | 0.711 (0.610–0.828) | <0.001 |
| Other GI cancer | 133 (1.0%) | 177 (1.3%) | 0.751 (0.601–0.940) | 0.005 |
| MACE | 950 (6.9%) | 1,111 (7.5%) | 1.070 (0.975–1.273) | <0.081 |
| Heart failure | 1,908 (13.8%) | 2,071 (15.0%) | 0.921 (0.870–0.976) | <0.001 |
| ESRD | 145 (1.1%) | 459 (3.3%) | 0.465 (0.385–0.561) | <0.001 |
| MAKE | 469 (3.4%) | 922 (6.7%) | 0.850 (0.760–0.952) | 0.005 |

# eTable 13. SGLT-2 Inhibitor and GLP-1 Receptor Agonist Drug Codes

## SGLT-2 Inhibitors

| **Drug Name** | **Brand Name(s)** | **RxNorm Code** |
| --- | --- | --- |
| Dapagliflozin | Farxiga | 1488564 |
| Empagliflozin | Jardiance | 1545653 |
| Canagliflozin | Invokana | 1373458 |
| Ertugliflozin | Steglatro | 1992672 |
| Bexagliflozin | Brenzavvy | 2627044 |

## GLP-1 Receptor Agonists

| **Drug Name** | **Brand Name(s)** | **RxNorm Code** |
| --- | --- | --- |
| Semaglutide | Ozempic, Wegovy, Rybelsus | 1991302 |
| Liraglutide | Victoza, Saxenda | 475968 |
| Dulaglutide | Trulicity | 1551291 |
| Exenatide | Byetta, Bydureon | 60548 |
| Tirzepatide | Mounjaro, Zepbound | 2601723 |
| Lixisenatide | Adlyxin | 1440051 |

# eTable 14. Outcome Definitions and ICD-10 Codes

| **Outcome** | **Definition** | **ICD-10/CPT Codes** |
| --- | --- | --- |
| Colon cancer (Primary) | Malignant neoplasm of colon | C18.0-C18.9, C19, C20 |
| Colectomy | Surgical removal of colon | CPT: 44140-44160, 44204-44212 |
| Other GI cancer | Upper GI, hepatobiliary malignancies | C15-C17, C22-C26 |
| MACE | MI, ischemic stroke, hemorrhagic stroke | I21-I22, I63, I61 |
| Heart failure | Heart failure diagnosis | I50.x |
| End-stage renal disease | ESRD or dialysis initiation | N18.6, Z99.2 |
| Major adverse kidney events | Composite kidney outcome | N18.4-N18.6, N17.x |
| Acute kidney injury | Acute renal failure | N17.x |
| All-cause mortality | Death event | Death record in EHR |
| Acute pancreatitis | Acute inflammation of pancreas | K85.x |
| Urinary tract infection | UTI diagnosis | N30.0, N39.0 |
| Inguinal hernia (negative control) | Groin hernia | K40.0-K40.9 |
| Appendicitis (negative control) | Appendix inflammation | K35-K37 |

# eTable 15. Propensity Score Matching Covariates

Complete list of covariates included in the propensity score model, assessed during the baseline period (before index event).

| **Category** | **Variables** |
| --- | --- |
| Demographics (3) | Age at index, Sex (Male/Female), Race (White, Black, Asian, Unknown) |
| Cardiovascular (6) | Essential hypertension (I10), Heart failure (I50), Ischemic heart disease (I20-I25), Chronic kidney disease (N18), Cerebrovascular diseases (I60-I69), Dyslipidemia (E78) |
| Other Disease (5) | Overweight and obesity (E66), BMI ≥30 kg/m², Nicotine Dependence, Family history of Colon cancer, Alcohol |
| Medications (8) | Biguanides (Metformin), Sulfonylureas, Alpha-glucosidase inhibitors, Thiazolidinediones, Insulin, DPP-4, Aspirin, NSAID |
| Laboratory Values (4) | BMI (continuous), BMI category (<30/≥30), HbA1c (continuous), HbA1c category (<7%/≥7%), Creatinine |

*All covariates assessed at baseline (before index event). SMD <0.1 for all variables after matching indicates adequate balance.*
